# The isoflavone puerarin exerts anti-tumor activity in pancreatic ductal adenocarcinoma by suppressing mTOR-mediated glucose metabolism

**DOI:** 10.18632/aging.203725

**Published:** 2021-12-04

**Authors:** Hengyue Zhu, Yanyi Xiao, Hangcheng Guo, Yangyang Guo, Youze Huang, Yunfeng Shan, Yongheng Bai, Xiangyang Lin, Hong Lu

**Affiliations:** 1 Key Laboratory of Diagnosis and Treatment of Severe Hepato-Pancreatic Diseases of Zhejiang Province, The First Affiliated Hospital of Wenzhou Medical University, Wenzhou 325000, China; 2 Department of Laboratory Medicine, The First Affiliated Hospital of Wenzhou Medical University, Wenzhou 325000, China; 3 Department of Laboratory Medicine, Wenzhou Hospital of Traditional Chinese Medicine, Wenzhou 325000, China; 4 Department of Hepato-Pancreato-Biliary Surgery, The First Affiliated Hospital of Wenzhou Medical University, Wenzhou 325000, China; 5 Center for Health Assessment, Wenzhou Medical University, Wenzhou 325000, China

**Keywords:** puerarin, pancreatic ductal adenocarcinoma, glucose metabolism, apoptosis, Akt/mTOR

## Abstract

Puerarin (8-(β-D-glucopyranosyl)-4′, 7-dihydroxyisoflavone), a natural flavonoid compound isolated from the traditional Chinese herb *Radix puerariae*, have been demonstrated has potential anti-tumor effects via induction of apoptosis and inhibition of proliferation. However, the effect and molecular mechanism of puerarin in pancreatic ductal adenocarcinoma (PDAC) remains unknown. In this study, the tumor-suppressive effects of puerarin were determined by both *in-vitro* and *in-vivo* assays. The effects of puerarin on the proliferation, apoptosis, migration and invasion of pancreatic cancer cells (PCCs), and tumor growth and metastasis in PDAC xenograft mouse model were performed. Puerarin treatment significantly repressed PCC proliferation. Puerarin induced the mitochondrial-dependent apoptosis of PCCs by causing a Bcl-2/Bax imbalance. Moreover, puerarin inhibited PCC migration and invasion by antagonizing epithelial-mesenchymal transition (EMT). In nude mouse model, PDAC growth and metastasis were reduced by puerarin administration. Mechanistically, puerarin exerted its therapeutic effects on PDAC by suppressing Akt/mTOR signaling. Importantly, puerarin bound to the kinase domain of mTOR protein, affecting the activity of the surrounding amino acid residues associated with the binding of the ATP-Mg^2+^ complex. Further studies showed that the inhibitory effects of puerarin on PCCs were abolished by a mTOR activator, indicating a crucial role of mTOR in anti-tumor effects of puerarin in PDAC. As a result, puerarin hindered glucose uptake and metabolism by downregulating the oxygen consumption rate (OCR) and the extracellular acidification rate (ECAR) dependent upon HIF-1α and glucose transporter GLUT1. Therefore, these findings indicated that puerarin has therapeutic potential for the treatment of PDAC by suppressing glucose uptake and metabolism via Akt/mTOR activity.

## INTRODUCTION

Pancreatic ductal adenocarcinoma (PDAC) is the most common exocrine pancreatic cancer seen clinically. It is the fourth-leading cause of cancer-related death in the United States, second only to colorectal cancer in gastrointestinal-related deaths [1]. According to the World Health Organization (WHO) GLOBOCAN database and the 2017 Global Burden of Disease Study, PDAC is the seventh leading cause of cancer deaths in men and women worldwide [2]. Surgical resection is the only possible cure. Unfortunately, only 15% to 20% of PDAC patients are eligible for a pancreatectomy due to the late discovery. However, even after complete resection, the prognosis of PDAC patients is poor. After resection margin-negative (R0) pancreaticoduodenectomy, the five-year survival rate of PDAC patients is about 30% for lymph node-negative and 10% for lymph node-positive patients [3, 4]. The median survival of patients with untreated and unresectable locally advanced PDAC is 8-12 months, while the median survival of patients with metastatic disease at presentation is only 3–6 months. Systemic chemotherapy can improve the survival rate of patients with locally advanced and metastatic PDAC. In today’s modern treatment era, the FOLF NO × regimen (fluorouracil + leucovorin, irinotecan, and oxaliplatin) has achieved the best outcome, but the median patient survival time is only 11.1 months [5]. New drugs, new drug targets, and new, more effective chemotherapy regimens are desperately needed in all settings.

Puerarin is a white crystal extracted from the roots of the kudzu plant or the kudzu vine. Its chemical name is 8-(β-D-glucopyranosyl)-4′,7-dihydroxyisoflavone, and its molecular formula is C_21_H_20_O_9_ [6]. Puerarin is the most abundant secondary metabolite, which was isolated from the rhizome of Pueraria lobata in the 1950s and is known as Asian ginseng. Since then, extensive research has been conducted on its pharmacological properties. Puerarin has various pharmacological effects, such as enhancing the circulatory system function, reducing myocardial oxygen consumption, decreasing blood sugar, and preventing hypertension and arteriosclerosis. Anti-liver toxicity, anti-inflammatory, expectorant, antipyretic, immunity-enhancing, antibacterial, and antiviral activities have also been demonstrated [7–9]. Its low toxicity and wide range of pharmacological effects have attracted the attention of domestic and foreign researchers. In recent years, the anti-cancer effect of puerarin has been widely studied. Many studies showed that puerarin had good anti-tumor activity in animal model and many cancer cell lines [10].

However, the role of puerarin in PDAC has not been studied in-depth and needs to be further explored. Therefore, in this study, we investigated the effects of puerarin on PDAC, and explored the underlying molecular mechanisms in various pancreatic cancer cell (PCC) lines *in vitro* and in a nude mouse xenograft model *in vivo.*

## MATERIALS AND METHODS

### Cell culture and drug treatment

**Figure 1:**
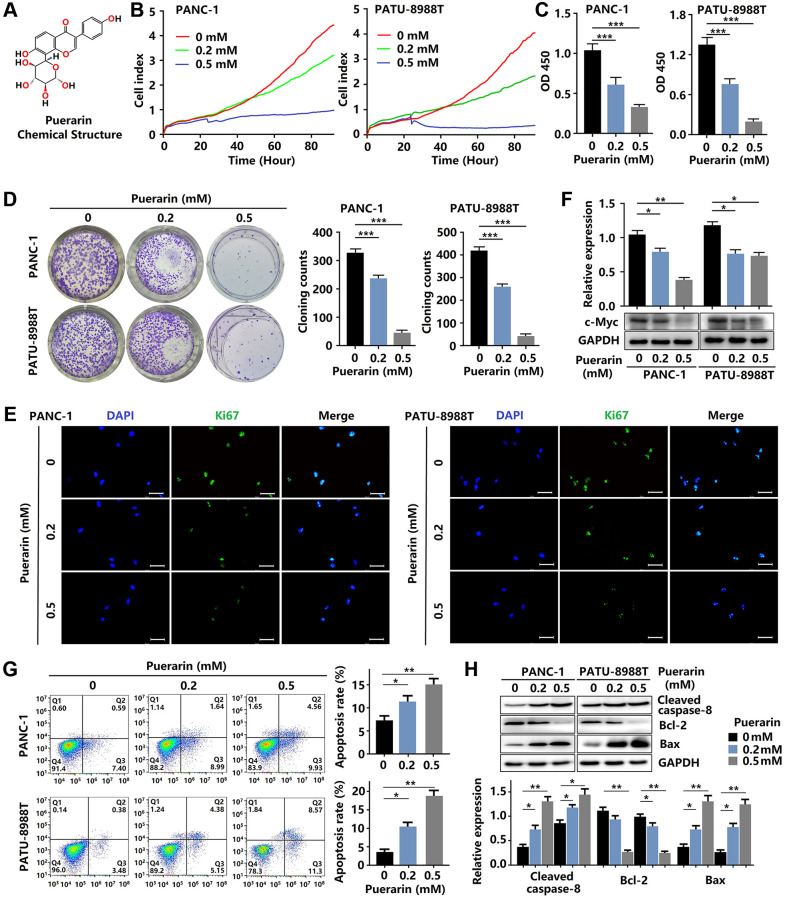
**Puerarin inhibits cellular proliferation and induces mitochondrial-dependent apoptosis in PDACs.** (**A**) The chemical structure of puerarin. (**B**) The growth of PDACs with or without puerarin treatment was determined by (RTCA). (**C**) The viability of PDACs was analyzed by the CCK-8 assay. (**D**) The proliferation of PDACs in different groups was analyzed by the colony formation assay. (**E**) Immunocytochemical staining of Ki67 in PDACs. Bar = 100 μm. (**F**) Protein expression of c-Myc in PDACs. (**G**) Flow cytometry analysis of cell apoptosis in PDACs with or without puerarin treatment. (**H**) Western blot analysis showing the expression of Cleaved caspase-8, Bcl-2, and Bax in PDACs. Data were presented as the mean ± standard deviation and were analyzed by one-way ANOVA with Bonferroni’s post-hoc test. ^*^p < 0.05, ^**^p < 0.01, and ^***^p < 0.001.

Human PCC lines PANC-1 and PATU-8988T were purchased from the Cell Bank of the Chinese Academy of Sciences (Shanghai, China). The cells were cultured in Dulbecco’s modified Eagle’s medium (DMEM, Invitrogen, Carlsbad, CA, USA) supplemented with 10% fetal bovine serum (FBS). The medium contained penicillin (100 U/mL) and streptomycin (100 μg/mL). FBS, penicillin and streptomycin were from Invitrogen. The cultured cells at a density of 1 × 10^6^ were initially plated in a 10-cm dish for 24 h. The culture medium was replaced with serum-free medium after 24 h. PDACs were treated with 0.2 and 0.5 mM puerarin (Figure 1A, puerarin chemical structure, CAS#: 3681-99–0, Purity: ≥98% by High Performance Liquid Chromatography, Yuanye Biotechnology, Shanghai, China) with or without MHY1485 (CAS#: 326914-06-1, MedChem Express, Monmouth Junction, NJ, USA).

### Cell counting kit-8 (CCK-8) assay

According to the manufacturer’s instructions, the CCK-8 assay kit (Dojindo, Shanghai, China) was used to detect the cell proliferation of PDACs. First, the cells were cultured in 6-cm dishes with fresh medium for 24 h. The cells in the logarithmic growth stage were inoculated into 96-well plates at a density of 5 × 10^3^ cells/ml. Then, the cells were treated with different concentrations of puerarin for 24 h. After that, 10 μl of CCK-8 medium and 10 μl of CCK-8 were added and the plates were incubated for another 4 h. The absorbance was measured at a wavelength of 450 nm using a microplate reader. Statistical analyses were performed using Stata statistical software (StataCorp LP). Each experiment was repeated thrice and the average value was taken as the final result.

### Flow cytometry analysis

The cells were serum-starved for 24 h and the medium was replaced with complete medium. PDACs were exposed to culture medium containing different concentrations of puerarin for 24 h, and cells in the standard control group were treated with dimethyl sulfoxide (DMSO, Sigma-Aldrich, St. Louis, MO, USA). After centrifugation to collect the cells, quantification of the apoptotic cells was performed using an Annexin V-FITC Apoptosis Detection Kit (Multisciences, Hangzhou, China) according to the manufacturer’s instructions. Cell apoptosis was assessed by flow cytometry (BD FACSVerse™, BD Biosciences, USA), and the results were analyzed using FlowJo (TreeStar, Ashland, OR, USA).

### Real-time cellular analysis (RTCA)

Cell proliferation was monitored by the xCELLigence RTCA MP System (ACEA Biosciences, San Diego, CA, USA) using 16-well E-Plates (ACEA Biosciences). The cells were seeded in triplicate at 5 × 10^3^ cells/well in the plates. For the RTCA experiments, the cells were treated with puerarin after reaching steady growth (24 h). Impedance was measured every 15 min over 96 h and represented as the cell index by the RTCA-integrated software of the xCELLigence System. The cell index was normalized to 1 at the time point of drug administration. From this data, real-time cell growth curves were generated with GraphPad Prism 7 (GraphPad Software, La Jolla, CA, USA).

### Transwell invasion assay

Transwell assays were performed using Transwell chambers (Costar, New York City, NY, USA) with Matrigel^®^ (BD Biosciences). After treatment with various concentrations of puerarin for 24 h, cell suspensions were prepared using ethylenediaminetetraacetic acid (EDTA) enzyme. The cells were resuspended in serum-free medium and transferred to the inner chamber (5 × 10^4^ cells per chamber). Complete medium was added to the outer chamber, and the plate was incubated in a CO_2_ incubator (37°C) for observation for 12 h. After carefully removing the non-migrating cells at the membrane site with a cotton swab, the cells were fixed with formaldehyde and stained with 0.1% crystal violet (Sigma), and quantification was performed by counting five random fields under the microscope (Leica Microsystems, Wetzlar, Germany). Each experiment was repeated three times.

### Colony formation assay

The cells were seeded into 6-well plates at 1 × 10^3^ cells per well and treated with puerarin 24 h later. After 24 h, the media was replaced with fresh media and cultured for 14 days. The colonies were then fixed with 2% formaldehyde and stained with 0.5% crystal violet. The number of colonies with ≥50 cells was counted under a microscope.

### Wound healing assay

PDACs were seeded in 6 well plates and maintained at 37°C for 24 h. The cells were scratched using a crystal pipette tip to make a linear gap. Next, the detached cells were washed away with phosphate-buffered saline (PBS) and different concentrations of puerarin were added. The cells were allowed to fill the gap, and after 24 h, images of the areas were captured using a microscope (Leica Microsystems).

### Immunocytochemical staining

Immunofluorescence staining was performed based on established protocols. PDACs with different treatments were grown on glass coverslips for 24 h. The cells were fixed with 4% formaldehyde and permeabilized with 0.1% Triton X-100 (Thermo Scientific, Waltham, MA, USA). Blocking was performed with 4% goat serum (Gibco, Thermo Fisher Scientific) in Dulbecco’s phosphate-buffered saline (DPBS; Invitrogen, Paisley, UK) for 1.5 h at 37°C, followed by incubation with the primary antibodies (Supplementary Table 1) at 4°C overnight. Next, the membranes were incubated in the appropriate second antibodies for 1 h at room temperature. At least three independent experiments for immunofluorescence staining were conducted.

### Western blot analysis

After treating the cells for 24 h, the cells in each group were collected and the total cellular protein was extracted. After separation by sodium dodecyl sulfate-polyacrylamide gel electrophoresis (SDS-PAGE), the proteins were transferred onto polyvinylidene fluoride (PVDF) membranes. The membranes were blocked with 5% non-fat milk for 1 h at room temperature and incubated overnight at 4°C with the primary antibodies (Supplementary Table 1). The membranes were washed three times in Tris-buffered saline with 0.1% Tween 20 (TBST) the following day and incubated with the second antibody (anti-rabbit IgG) at room temperature for 1 h. After the membranes were rinsed, the protein expression levels were detected by enhanced chemiluminescence (ECL) and visualized by autoradiography. GAPDH was used as the internal reference protein.

### Glucose metabolism assay

The oxygen consumption rate (OCR) and extracellular acidification rate (ECAR) in intact cells were measured using a Seahorse XF96 Analyzer (Agilent Technologies, Santa Clara, CA, USA). Concisely, 1 × 10^4^ PDACs were seeded into 96-well seahorse cell culture plates and incubated overnight at 37°C. Then the cells were pretreated with different concentrations of puerarin for 24 h. For OCR detection, the compounds were added as follows: oligomycin (2.5 μM), carbonyl cyanide 4-(trifluoromethoxy), phenylhydrazone (FCCP, 2 μM), rotenone (0.25 μM). The ECAR was evaluated after the sequential injection of glucose (10 mM), oligomycin (1 μM), and 2-Deoxy-D-glucose (2-DG, 50 mM). At the end of the experiment, overall OCR and ECAR curves were normalized to protein concentrations and plotted using Wave software (Agilent Technologies).

### Nude mouse tumorigenicity

BALB/c nude mice (6–8 weeks old, Wenzhou Medical University Experimental Animal Center, Wenzhou, China) were used to establish the nude mouse xenograft model. All mice were housed under controlled conditions (temperature, 21–23°C; 12 h light/dark cycle; 55% humidity). PANC-1 cells (3 × 10^6^) in 0.2 ml PBS were subcutaneously injected into the right thighs of nude mice, and randomly divided twelve mice into two groups (*n* = 6 in each group). Puerarin was solubilized in normal saline buffer. Mice in the experimental group was injected puerarin (50 mg/kg) by oral gavage every three days for one month. The control group received normal saline injections by oral gavage for one month [11]. Tumor formation in the nude mice was monitored for 30 days. The mice were deeply anesthetized with sodium pentobarbital and euthanized by cervical dislocation [12–14]. The tumor size was calculated according to the standard formula: tumor volumes (cm^3^) = (the longest diameter) × (the shortest diameter)^2^ × 0.5.

This animal study was approved by the Institutional Animal Care and Use Committee of Wenzhou Medical University, China. The animal experiments were conducted according to all regulatory and institutional guidelines for animal welfare (National Institutes of Health Publications, NIH Publications No. 80-23) [15].

### Molecular docking

Molecular docking was performed as previously described [16], Puerarin and mTOR were rigidly docked, and the docking results were analyzed by PyMOL software. The puerarin molecule was downloaded from Pubchem, and the molecular energy was optimized through Chem 3D Ultra Software (8.0.3 version, Cambridge-Soft, MA, USA). The crystal structure of mTOR was downloaded from the Protein Structure Database (Protein Data Bank, PDB) (http://www.rcsb.org/pdb/), and the protein was processed by Autodock (MGLTools-1.5.6) to remove water molecules and hydrogenate and to add volume.

### Histopathological analysis

Tumor specimens from the animals were paraffin-embedded and cut into 4-μm-thick sections. Standard hematoxylin-eosin (H&E) staining (Biyuntian, Hangzhou, China) was performed. According to a previous method, immunohistochemical (IHC) analysis was conducted under a microscope [15]. IHC staining was performed using the following primary antibodies (Supplementary Table 1). Two independent investigators semi-quantitatively assessed all samples in a blinded manner.

### Database analysis

The correlation between AKT and mTOR expression and the activity of KRAS, TP53, CDKN2A, and SMAD4 were evaluated in the GEPIA 2 database website (http://gepia2.cancer-pku.cn/#analysis).

### Statistical analysis

The data are expressed as the mean ± standard deviation for the *in vitro* and *in vivo* experiments. All statistical analyses were performed using GraphPad Prism statistical analysis software (version 8.0, GraphPad Software, Inc., LaJolla, CA, USA). Statistical comparisons were made with a two-sided *t*-test. One-way analysis of variance (ANOVA) with Bonferroni’s post-hoc test was used when more than two groups were present. Statistical significance was indicated by a *P*-value of <0.05.

### Ethics statement

Animal experiments were approved by the Committee for Animal Experiments at Wenzhou Medical University.

## RESULTS

### Puerarin inhibits PCC proliferation and induces mitochondria-mediated apoptosis

To investigate the effect of puerarin on PCC proliferation, the RTCA, CCK-8, and colony formation assays were performed. As shown in Figure 1B, 1C, as expected, puerarin treatment (0.2 and 0.5 mM) significantly inhibited the growth of PDACs in concentration- and time-dependent manners. The CCK-8 assay results of the PDACs confirmed the concentration-dependent inhibition of cell growth by puerarin (Figure 1B, 1C). Puerarin also significantly reduced colony formation in the PDACs (Figure 1D). To investigate the effect of puerarin on cell proliferation, we used immunofluorescence staining for the Ki67 marker expressed by proliferating cells. The level of Ki67 protein varied with the cell cycle and was higher in the G2/M phase and lower in the G0/G1 phase [17]. In the PDACs, we observed a decrease in Ki67 protein expression in both the PANC-1 and PATU-8988T cells treated with puerarin (Figure 1E) compared to the control cells. In addition, puerarin reduced the expression of proliferation-related protein c-Myc (Figure 1F). Therefore, the above results suggest that puerarin inhibited PCC proliferation in concentration- and time-dependent manners.

Then, we evaluated the effects of puerarin on PCC apoptosis by flow cytometry analysis. Puerarin treatment significantly increased the proportion of apoptotic and necrotic cells (Figure 1G). Further studies showed increased caspase-8 in both the PANC-1 and PATU-8988T cells treated with puerarin (Figure 1H). Apoptosis in cancer cells depends upon the dynamic equilibrium of Bax and Bcl-2 expression [18]. Puerarin was observed to increase Bax expression and decrease Bcl-2 expression (Figure 1H). These results suggest that puerarin induced the death receptor- and mitochondrial-mediated apoptosis of PCCs.

### Puerarin inhibits the migration and invasion of PCCs by antagonizing epithelial-mesenchymal transition

**Figure 2:**
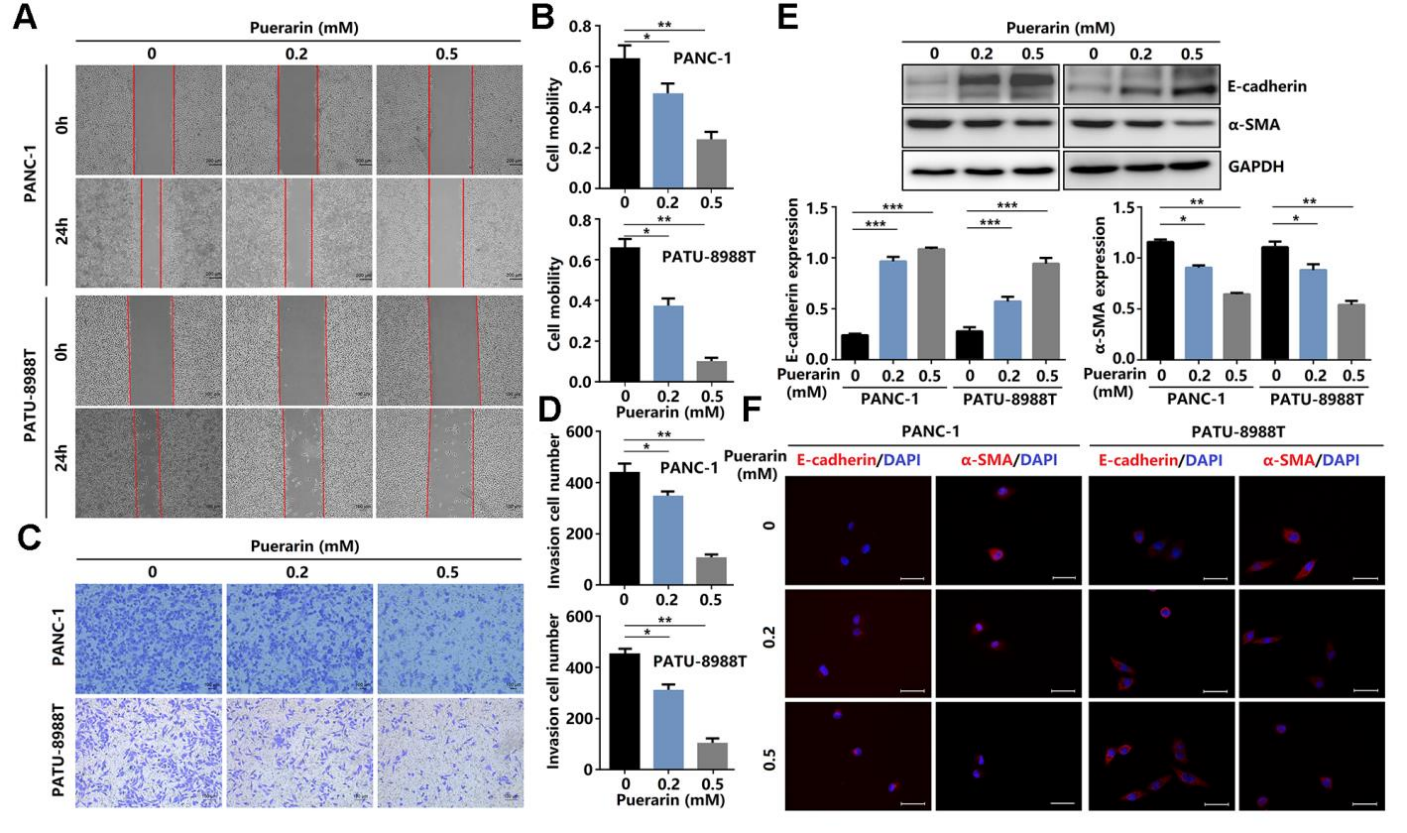
**Puerarin inhibits PCC invasion and migration by antagonizing the Slug-E-cadherin axis.** (**A**, **B**) The effect of puerarin on the migrated rate of PDACs was determined by the wound healing assay. (**C**, **D**) The effects of puerarin on the invading number of PDACs were analyzed by the transwell chamber assay. (**E**) Western blot analysis showing the expression of E-cadherin and α-SMA in puerarin-treated PDACs. (**F**) Immunocytochemical staining of E-cadherin and α-SMA in puerarin-treated PDACs. Bar = 25 μm. The data are presented as the mean ± standard deviation and were analyzed by one-way ANOVA with Bonferroni’s post-hoc test. ^*^p < 0.05, ^**^p < 0.01, and ^***^p < 0.001.

Enhanced cell migration and invasion abilities underlie PCC metastasis mechanisms, resulting in poor prognosis [19]. Here, puerarin reduced the migration rate of PDACs as determined by the scratch wound assay (Figure 2A, 2B) and the effect was concentration-dependent. Also, puerarin treatment significantly inhibited the numbers of invading PDACs detected by the transwell assay (Figure 2C, 2D). Further studies showed that puerarin decreased the protein level of α-SMA in PDACs and increased the E-cadherin protein level (Figure 2E). Immunofluorescence analysis revealed the downregulated expression of α-SMA and the increased expression of E-cadherin after puerarin treatment (Figure 2F). In general, these results suggest that puerarin inhibited PCC migration and invasion by inhibiting EMT and tumor mammosphere formation.

### Puerarin suppresses PDAC growth *in vivo*

**Figure 3:**
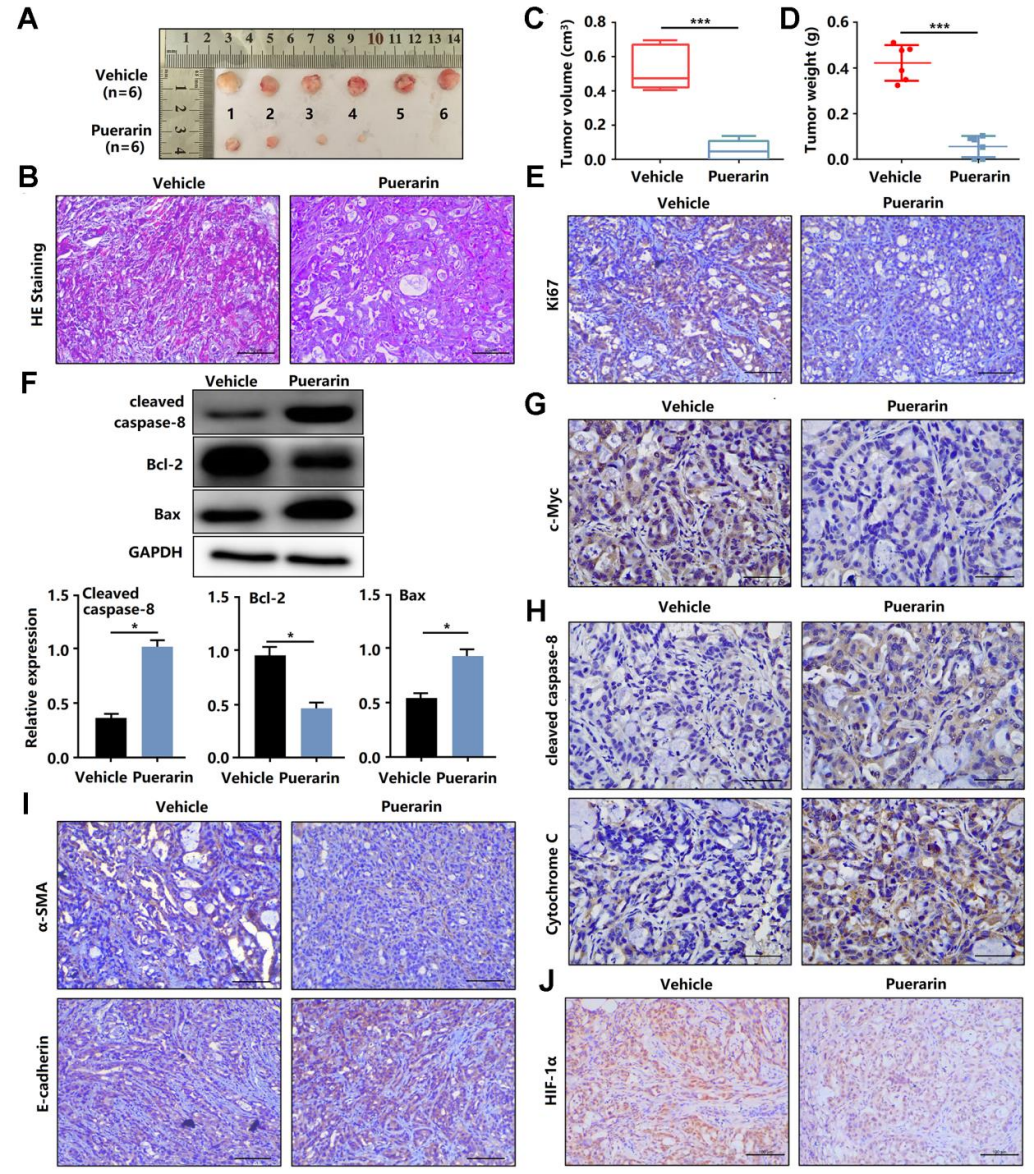
**Puerarin inhibits the tumor growth and metastasis of PDAC in the animal xenograft model.** (**A**) Effects of puerarin on morphologic changes in the experimental groups. (**B**) Pathological results of H&E staining for PDAC in tissues of the model group. Bar = 50 μm. (**C**) Effect of puerarin on the volume of tumors in the animal xenograft model. (**D**) Effects of puerarin on tumor weight. (**E**) IHC staining for Ki67 in the puerarin-treated model. Bar = 50 μm. (**F**) IHC staining for c-Myc in the puerarin-treated model. Bar = 50 μm. (**G**) Protein expression of Cleaved caspase-8, Bax and Bcl-2 in PDACs in different groups. (**H**) IHC staining for Cleaved caspase-8 and cytochrome C in the puerarin-treated model. Bar = 50 μm. (**I**) IHC staining for E-cadherin and α-SMA in the puerarin-treated model. Bar = 50 μm. (**J**) IHC staining for HIF-1α in the puerarin-treated model. Bar = 50 μm. The data are presented as the mean ± standard deviation, and were analyzed by a two-sided Student’s *t*-test. ^*^p < 0.05 and ^***^p < 0.001.

To determine the anti-cancer effects of puerarin *in vivo*, nude mice were injected with PANC-1 cells and then administrated puerarin or DMSO as a control. Figure 3A shows the morphology of tumor xenografts changes in the experimental group after puerarin treatment. The pathological results in the PDAC model tissue were shown by H&E staining (Figure 3B). We found that the administration of puerarin significantly reduced tumor volume and weight (Figure 3C, 3D). In addition, puerarin administration downregulated the expression of Ki67 and c-Myc (Figure 3E, 3F). Moreover, puerarin upregulated the expression of Cleaved caspase-8 and Bax, and decreased Bcl-2 expression (Figure 3G, 3H), suggesting that puerarin inhibited PCC proliferation and induced death receptor- and mitochondrial-mediated apoptosis. To assess whether puerarin inhibited PDAC migration, we examined the expression of EMT process-related proteins. The results showed that puerarin decreased α-SMA expression and increased E-cadherin expression (Figure 3I). It also reduced the expression of c-Myc, an oncoprotein associated with tumor progression and chemoresistance (Figure 3F) [20–22]. Hypoxia is usually observed in PDAC and some other solid tumors. HIF-1α protein, a key regulator of the hypoxia response, was found to accumulate in PDAC tissues. Several studies have shown that hypoxia was an independent predictor of poor prognosis [23]. We observed the downregulation of HIF-1α protein after puerarin treatment (Figure 3J). In summary, these data suggest that puerarin inhibited the growth of PDAC in a mouse xenograft model.

### Puerarin reduces the activity of Akt/mTOR signaling *in vitro* and *in vivo*

**Figure 4:**
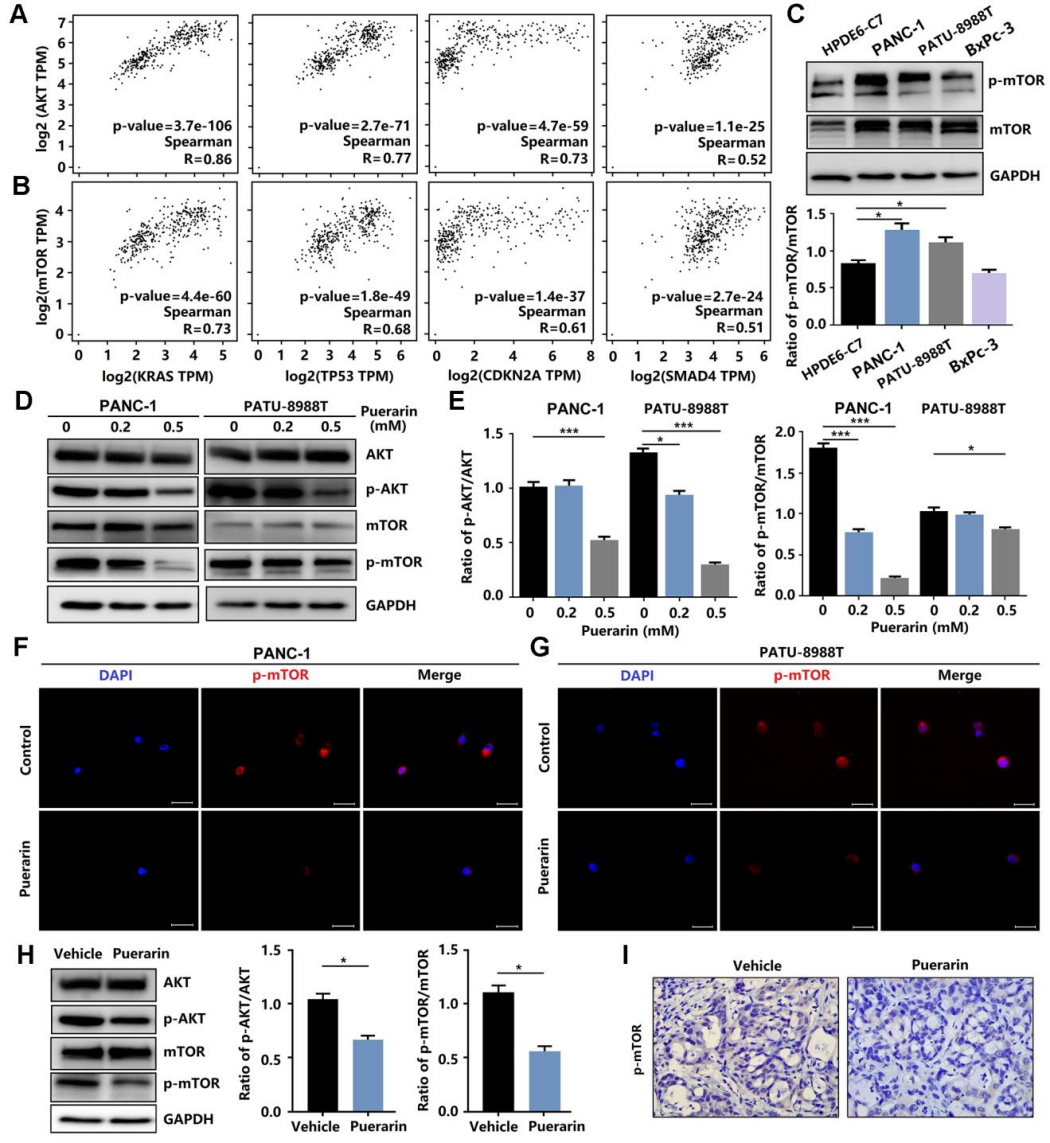
**Puerarin inhibits the activation of Akt/mTOR signaling *in vitro* and *in vivo*.** (**A**) The correlation between the expression of Akt and the activity of KRAS, TP53, CDKN2A, and SMAD4 in the GEPIA 2 database was evaluated. (**B**) The correlation between the expression of mTOR and the activity of KRAS, TP53, CDKN2A, and SMAD4 in the GEPIA 2 database was evaluated. (**C**) The expression and phosphorylation of mTOR in normal pancreatic ductal cells (HPDE6-C7) and PCCs (PANC-1, PATU-8988T, and BxPc-3). (**D**, **E**) Western blot analysis showing the expression and phosphorylation of Akt and mTOR in PDACs with or without puerarin treatment. (**F**, **G**) Immunocytochemical staining of mTOR in PDACs. Bar = 50 μm. (**H**) Western blot analysis showing the expression and phosphorylation of Akt and mTOR in the puerarin-treated animal xenograft model. (**I**) IHC staining for mTOR in the puerarin-treated model. Bar = 50 μm. The data are presented as the mean ± standard deviation and were analyzed by one-way ANOVA with Bonferroni’s post-hoc test and two-sided Student’s *t*-test. ^*^p < 0.05, ^**^p < 0.01, and ^***^p < 0.001.

Anti-cancer effects involve many mechanisms, including oxidative stress, intrinsic and extrinsic mechanisms, as well as the survivin, PI3K/Akt/mTOR, SHH [24], Nrf2/Keap1 [25], inflammation, and autophagy pathways [26]. Studies have shown that the signal transduction pathway mediated by phosphatidylinositol 3 kinase (PI3K) was closely related to cancer occurrence. Many downstream molecules make up the PI3K/Akt signal pathway, including mTOR, one of the more important targets of rapamycin. mTOR signaling plays a crucial role in cell growth, protein translation, autophagy, and metabolism [27]. In Pancreatic tissue, including PAAD (Pancreatic adenocarcinoma) Tumor, PAAD normal and pancreas, the activation of mTOR was associated with gene mutations, including KRAS, TP53, CKDN2A, and SMAD4, resulting in tumor development (Figure 4A, 4B). We also found that these PCCs exhibited heterogeneous PI3K/Akt/mTOR pathway activation at the protein level (Figure 4C). In this study, we investigated the effect of puerarin on mTOR activity in PDACs. We found that puerarin suppressed the mTOR signaling pathway (Figure 4D, 4E), suggesting that mTOR may be a target of puerarin. Puerarin-induced the downregulation of phosphorylated mTOR expression in PDACs (Figure 4F, 4G). The *in vitro* experiments confirmed that puerarin inhibited the overexpression of mTOR in PDAC tissues (Figure 4H, 4I).

### Puerarin binds to the kinase domain of mTOR protein to inhibit protein activity

**Figure 5:**
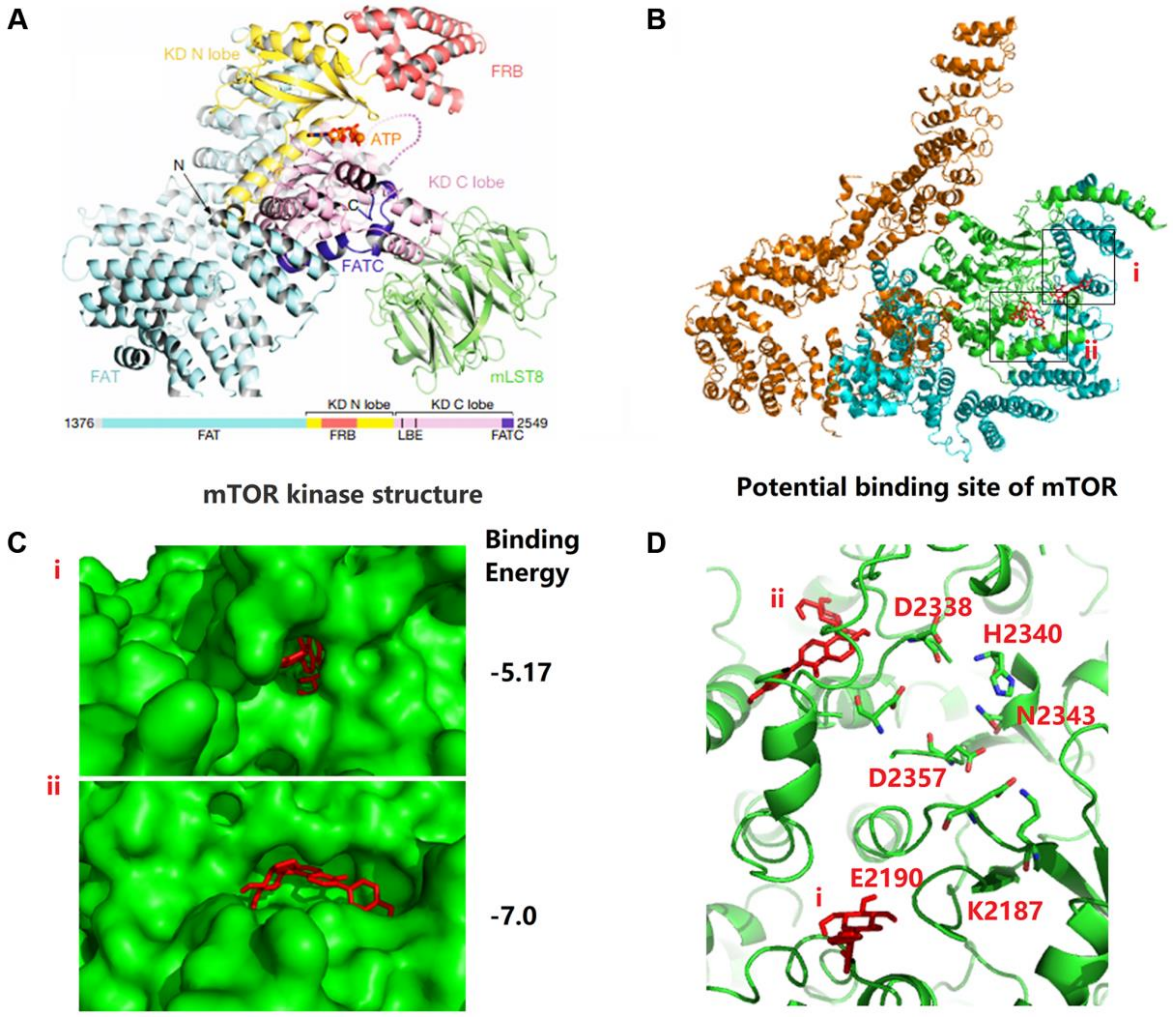
**Puerarin binds to the kinase domain of mTOR protein to inhibit protein activity.** (**A**) The kinase domain (KD, green cartoon) areas of mTOR. (**B**, **C**) The possible binding sites of puerarin and mTOR including two structural regions i and ii, and the binding energy is −5.17 and −7.0, respectively. (**D**) There are many ATP-Mg complex binding-related amino acid residues around the i and ii binding sites.

To further analyze the biochemical pathways of puerarin affecting mTOR protein, we used Autodock (MGLTools-1.5.6) to rigidly dock puerarin with the FAT domain (blue cartoon) and the kinase domain (KD, green cartoon) areas of mTOR (Figure 5A). We found that the possible binding sites of puerarin and mTOR included two structural regions i and ii (Figure 5B, 5C), with binding energies of −5.17 and −7.0, respectively. Figure 5D shows that there were many ATP-Mg complex binding-related amino acid residues around the i and ii binding sites. Once puerarin binds to the i and ii sites on mTOR protein, it may affect the activity of the above amino acid residues, and then affect mTOR activation activity.

### Activated mTOR signaling eliminates puerarin-mediated anti-tumor effects

**Figure 6:**
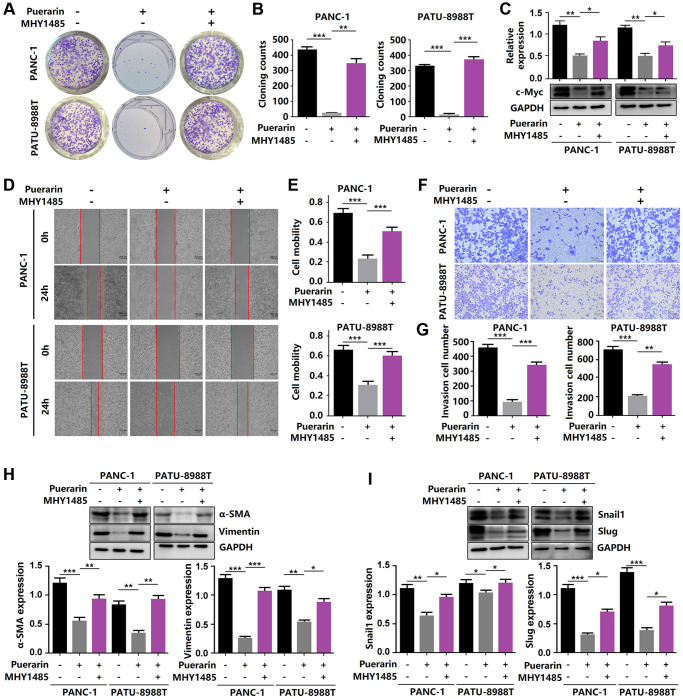
**Reactivation of mTOR reverses the anti-tumor effects of puerarin.** (**A**, **B**) The proliferation of puerarin-treated PDACs with or without MHY1485 treatment was analyzed by the colony formation assay. (**C**) Protein expression of c-Myc in puerarin-treated PDACs with or without MHY1485 treatment. (**D**, **E**) The migration ability of puerarin-treated PDACs in different groups was determined by the wound healing assay. (**F**, **G**) The invasion ability of puerarin-treated PDACs was analyzed by the transwell chamber assay. (**H**) Protein expression of vimentin and α-SMA in puerarin-treated PDACs. (**I**) Protein expression of Snail1 and Slug in puerarin-treated PDACs. The data are presented as the mean ± standard deviation and were analyzed by one-way ANOVA with Bonferroni’s post-hoc test and two-sided Student’s *t*-test. ^*^p < 0.05, ^**^p < 0.01, and ^***^p < 0.001.

Given the anti-tumor effect of puerarin on PDAC by inhibiting mTOR signal transduction, we next investigated whether activated mTOR signal transduction influenced this effect of puerarin. In the PANC-1 and PATU-8988T cells, we used MHY1485, a significant cell permeability mTOR activator to activate the mTOR pathway, targeting the ATP domain mTOR. The activation of mTOR signaling eliminated the anti-proliferative effect of puerarin (Figure 6A–6C). Using the transwell and wound healing assays, we demonstrated that MHY1485 treatment increased the invasion and migration rates of the PDACs (Figure 6D–6G). Thus, activated mTOR signaling eliminated puerarin-mediated EMT suppression, as shown by the increased expression of α-SMA, vimentin, Snail1, and Slug (Figure 6H, 6I). These findings confirmed that mTOR signaling played a crucial role in the anti-tumor effect of puerarin in PDAC.

### Puerarin inhibits mTOR-mediated glucose metabolism in PCCs

**Figure 7:**
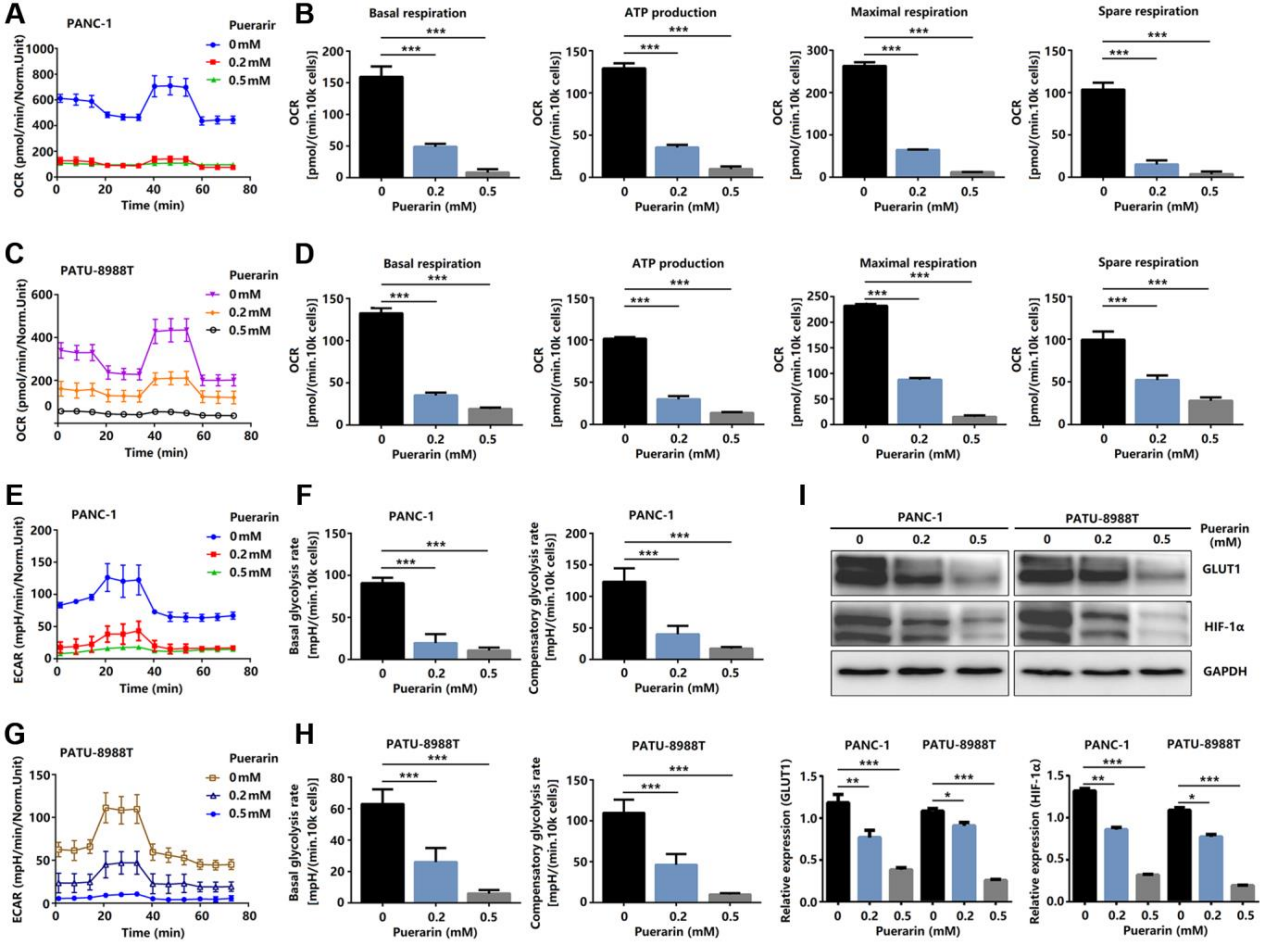
**Puerarin controls glucose metabolism in PCCs.** (**A**–**D**) Glucose metabolism assay showing downregulated levels of OCR, basal respiration, spare respiration, maximal respiration, and ATP production in puerarin-treated PDACs. (**E**–**H**) Glucose metabolism assay showing reduced ECAR, basal glycolysis, and compensatory glycolysis in puerarin-treated PDACs. (**I**) Protein expression of GLUT1 and HIF-1α in puerarin-treated PDACs. The data are presented as the mean ± standard deviation and analyzed by one-way ANOVA with Bonferroni’s post-hoc test and two-sided Student’s *t*-test. ^*^P < 0.05, ^**^P < 0.01, and ^***^P < 0.001.

To satisfy the need for rapid proliferation, tumor cells need more energy, so the process of bioenergy metabolism targeting tumor cells is a new therapeutic strategy to inhibit the growth of tumor cells [28]. A bioenergy analyzer was used to measure the corresponding OCR and ECAR, and the effects of external factors on mitochondrial uptake and glycolysis were analyzed statistically. The primary respiration, ATP production, maximum respiration, and spare respiration of cells treated with puerarin decreased significantly (Figure 7A–7D), indicating that puerarin inhibited the energy metabolism of the mitochondria. The glycolysis of the tumor cells treated with puerarin was significantly inhibited (Figure 7E, 7G). The results showed that the basal glycolysis rate and the compensatory glycolysis rate decreased significantly (Figure 7F, 7H). Further studies showed that GLUT1 and HIF-1α protein expression was inhibited (Figure 7I). Considering the close connection between puerarin and the mTOR pathway, our research results indicate that puerarin may regulate downstream GLUT1 through the mTOR pathway and affect tumor cell metabolism.

## DISCUSSION

Puerarin has certain anti-cancer effects in a variety of tumors. However, its role in PDAC is still poorly understood. In this study, we showed that puerarin treatment significantly repressed the proliferation of PCCs in concentration- and time-dependent manners. In addition, puerarin induced the mitochondrial-dependent apoptosis of PCCs by causing a Bcl-2/Bax imbalance. Moreover, puerarin inhibited the migration and invasion of PCCs by antagonizing EMT. In the nude mouse model, PDAC growth and metastasis were also reduced by puerarin administration. Thus, these *in vitro* and *in vivo* results indicate that puerarin exerted effective protection against PDAC. Previous studies have shown that puerarin impeded cell growth, blocked the cell development in the G0/G1 cell cycle phase, induced apoptosis in bladder cancer cells through the mTOR/p70 S6K signaling pathway, and suppressed cell growth and migration in human papillomavirus (HPV)-positive cervical cancer cells by inhibiting the PI3K/mTOR signaling pathway [29, 30]. In addition, puerarin 6’-O-xyloside, an analog of puerarin, suppressed hepatocellular carcinoma by regulating proliferation, stemness, and apoptosis by inhibiting PI3K/Akt/mTOR [31]. However, the anti-tumor effect and molecular mechanism of puerarin in PDAC remains unknown. Here, we identified effective protection against PDAC by puerarin and showed that the Akt/mTOR signaling pathway played an important role in the anti-tumor effect of puerarin.

mTOR protein kinase plays a key role in organizing the cellular and body physiology of all eukaryotes. In the two and a half years since its discovery, mTOR has been shown to be the central node in the network that controls cell growth. In this way, it integrates information about the availability of energy and nutrients to coordinate the synthesis or decomposition of new cellular components. The dysregulation of this basic signal transduction pathway can disrupt cellular homeostasis and may aggravate the overgrowth of cancer and pathology related to aging and metabolic diseases [32]. Although mTOR kinase itself is rarely mutated in cancer, it is easily hijacked by upstream oncogenic nodes, including those in the PI3K/Akt pathway and the MAPK pathway driven by Ras. As a result, mTOR signaling is active in as many as 80% of human cancers. In this case, mTOR signaling plays a key role in maintaining the growth and survival of cancer cells [33]. Cancer patients with acquired drug resistance have a poor prognosis, which prompted us to explore the vulnerability of cancer cells that are resistant to chemotherapy. The mTOR pathway is located downstream of the phosphoinositide 3-kinase (PI3K) and Akt pathway regulated by the phosphatase and tensin homolog (*PTEN*) tumor suppressor gene [34]. Inhibition of the mTOR pathway can inhibit tumor progression at multiple levels. In terms of mechanism, puerarin exerts a therapeutic effect on PDAC by inhibiting Akt/mTOR signal transduction activity, as shown by a decrease in phosphorylation and nuclear transcription. Further studies showed that the small molecule activator of mTOR, MHY1485, eliminated the puerarin-mediated inhibition of PCC proliferation and apoptosis induction. Viewing mTOR as a widespread driver of therapeutic resistance suggests considerable hope for targeting cancer drug resistance using mTOR inhibitors [35]. Significantly, puerarin inhibited the phosphorylation of mTOR, the downstream expression of GLUT1 and HIF-1α, and the glucose metabolism of PCC. In PDAC, even under normoxia, glycolysis is the primary energy source for cancer cell proliferation, invasion, migration, and metastasis [36]. We found that puerarin hindered glucose uptake and metabolism by downregulating the OCR and ECAR levels that depend upon HIF-1α and the glucose transporter GLUT1. Therefore, these findings indicate that puerarin has the therapeutic potential to treat PDAC by inhibiting the energy metabolism of tumor cells. Puerarin inhibited glucose uptake and metabolism by reducing the OCR and the ECAR dependent upon HIF-1α and glucose transporter GLUT1. Further studies showed that the mTOR small molecule activator MHY1485 could eliminate the puerarin-mediated inhibition of PCC proliferation and induction of apoptosis. Therefore, these findings suggest that puerarin has therapeutic potential for PDAC by inhibiting Akt/mTOR activity. The limitation of our study was that we did not explore the specific target of puerarin in the mTOR signal pathway, which needs further study.

In response to the increasing interest in drug development, researchers have actively tried to develop new treatment strategies, including neoadjuvant chemotherapy for patients with resectable or marginally resectable incremental cancers, multi-drug combination chemotherapy for patients with advanced PDAC, and new complex drugs or immuno-oncology drugs for PDAC patients with specific gene mutations.

Bax and Bak are two pro-apoptotic proteins with similar functions in the Bcl-2 family. Because of their essential role as effectors of mitochondrial outer membrane permeability (MOMP), Bcl and Bak are the portals of apoptosis in mitochondria, an essential step in the process of dependent apoptosis [37]. We observed an imbalance in the Bcl-2/Bax ratio after puerarin treatment, which indicated that puerarin could induce the mitochondria-dependent apoptosis of PCCs.

EMT is a cellular process in which epithelial cells acquire a mesenchymal phenotype and behavior after epithelial downregulation. The cells then exhibit fibroblast-like morphology and cellular structure and increase their ability to migrate. Also, these now-migrating cells are usually invasive [38]. Metastasis-related events are the leading cause of cancer-related death, and circulating tumor cells (CTCs) play a crucial role in metastatic recurrence. The EMT marker expressed in CTCs is closely related to poor clinical results. As mentioned in previous studies, puerarin inhibits migration and invasion by antagonizing EMT [39]. We studied the effects of puerarin on PCC proliferation, apoptosis, migration, and invasion, tumor growth, and metastasis in a PDAC xenograft mouse model. In the nude mouse model, the use of puerarin reduced the growth and metastasis of PDAC.

The limitation was that this study did not thoroughly explore the specific targets of puerarin acting in the mTOR signaling pathway. At the same time, our study used two cell lines, PANC-1 and PATU-8988T, so they could not fully cover the entire range of the tumor. More importantly, a genetic approach to exploring the association between mTOR signaling and the anti-tumor effects of puerarin needs to be implemented.

**Figure 8:**
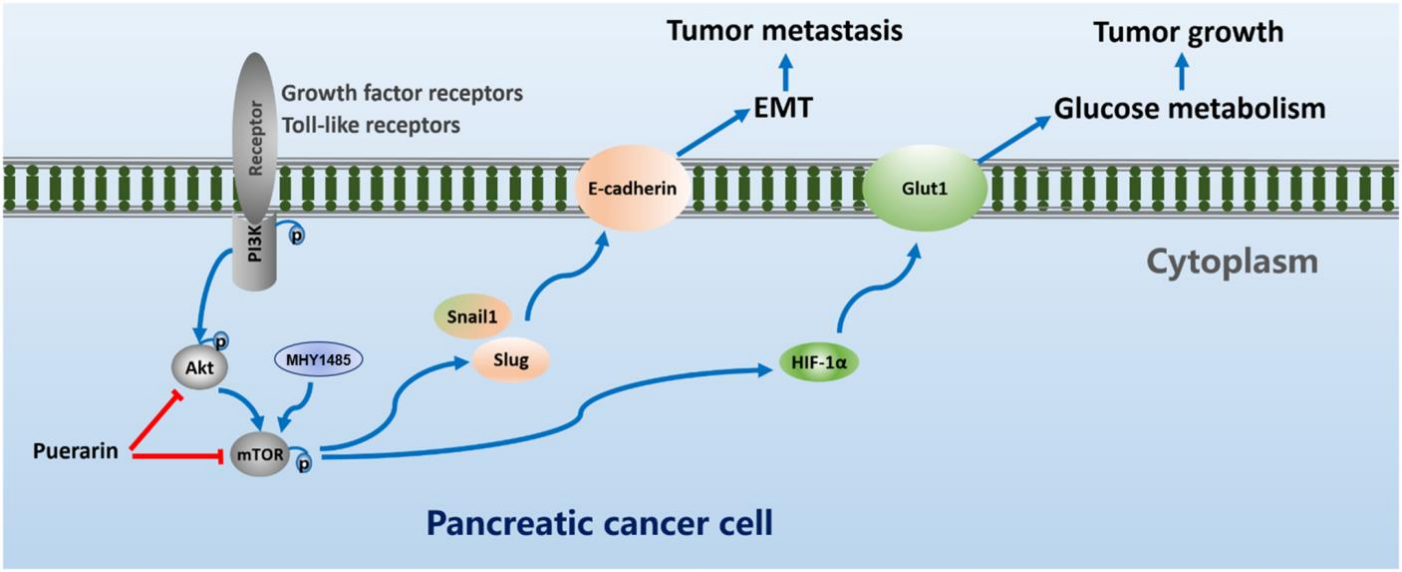
Puerarin suppresses oncogenesis and progression of PDAC via suppressing Akt/mTOR activity.

In conclusion, our results revealed that puerarin had a clear function in pancreatic cancer. It inhibited tumor cell proliferation and migration. Interestingly, our results suggest that the mTOR signaling pathway may play an important role in the anti-tumor process of puerarin. The process also involves downregulation of the OCR and the ECAR dependent upon HIF-1α and the glucose transporter GLUT1 to inhibit glucose uptake and metabolism. In addition, puerarin inhibited the migration and invasion of PCCs by antagonizing the EMT. In the nude mouse model, puerarin inhibited the growth and metastasis of PDAC. Further studies showed that MHY1485, a small molecule activator of mTOR, could block the puerarin-mediated effect of inhibiting PCCs proliferation and inducing PCCs apoptosis (Figure 8). Therefore, puerarin has the potential to treat PDAC by inhibiting Akt/mTOR activity.

## Supplementary Materials

Supplementary Table
