# Supplementary material for: The isoflavone puerarin exerts anti-tumor activity in pancreatic ductal adenocarcinoma by suppressing mTOR-mediated glucose metabolism
Source: Aging (Albany NY). 2021 Dec 4;13(23):25089–105. doi: 10.18632/aging.203725 (PMC8714170; doi:10.18632/aging.203725)
Supplement: Supplementary Table [file aging-13-203725-s001.pdf]

SUPPLEMENTARY TABLE

Supplementary Table 1. Primary antibodies in this study.

Reagent or Resource	Source	Identifier
Rabbit polyclonal anti-caspase8	Proteintech	Cat# 13423-1-AP
Rabbit polyclonal anti-Bcl-2	Proteintech	Cat# 12789-1-AP
Rabbit polyclonal anti-Bax	Proteintech	Cat# 50599-2-Ig
Rabbit monoclonal anti-α-SMA	ABclonal	Cat# A17910
Rabbit monoclonal anti-E-Cadherin	Abcam	Cat# ab231303
Rabbit polyclonal anti-Vimentin	Abcam	Cat# ab45939
Rabbit monoclonal anti-c-Myc	Abcam	Cat# ab32072
Rabbit polyclonal anti-Snail1	Proteintech	Cat# 13099-1-AP
Rabbit monoclonal anti-mTOR	Abcam	Cat# ab32028
Rabbit polyclonal anti-p-mTOR	Abcam	Cat# ab131538
Rabbit monoclonal anti-Ki67	Abcam	Cat# ab16667
Rabbit polyclonal anti-GAPDH	Proteintech	Cat# 10494-1-AP
Rabbit polyclonal anti-p-AKT	Abcam	Cat# ab38449
Rabbit polyclonal anti-AKT	Abcam	Cat# ab8805
Rabbit polyclonal anti-GLUT1	Affinity	Cat# AF0173
Rabbit polyclonal anti-SLUG	Abcam	Cat# 27568
Rabbit monoclonal anti-HIF1-α	Abcam	Cat# ab1
Rabbit polyclonal anti-Cytochrome C	Abcam	Cat# ab90529
